# Ventriculoperitoneal Shunt-Associated Ascites: A Case Report

**DOI:** 10.7759/cureus.8634

**Published:** 2020-06-15

**Authors:** Saud E Suleiman, Anastasia Tambovtseva, Elena Mejery, Ziad Suleiman, Ziad Alaidy

**Affiliations:** 1 Gastroenterology, Florida State University (FSU) College of Medicine, Daytona Beach, USA; 2 Advanced Gastroenterology, Halifax Medical Center, Daytona Beach, USA; 3 Internal Medicine, Ocala Regional Medical Center, Ocala, USA; 4 Internal Medicine, Medical University of the Americas, Jackson, USA; 5 Biology, University of Florida, Gainesville, USA; 6 Breast Cancer Research, Johns Hopkins Hospital, Baltimore, USA

**Keywords:** ventriculoperitoneal shunt, ascites, hydrocephalus, csf, peritoneal fluid, ventriculoatrial shunt

## Abstract

A ventriculoperitoneal shunt is a commonly performed procedure that is used to relieve the increased intracranial pressure in patients with hydrocephalus. VP shunt placement is an invasive procedure and carries many complications. Besides common complications like infections or mechanical obstruction, VP shunt has been found to be associated with the development of ascites in some patients. VP shunt-associated ascites is a very rare complication and only a few cases have been reported in the literature, most of which were in the pediatric population, while adult VP shunt-associated ascites was even rarer.

The patient in this case is a 32-year-old female who presented with ascites of unclear etiology. She had a history of VP shunt placement shortly after birth due to central nervous system (CNS) malformation (agenesis of the corpus callosum). Liver pathology, infection, and malignancy were ruled out as potential causes, and ascites was determined to be due to VP shunt drainage.

The exact mechanism of development of ascites in these patients is not fully understood and needs to be investigated further to optimize preventative and therapeutic options.

## Introduction

Hydrocephalus is a condition that results from increased intracranial pressure. It can be caused by various conditions, including intracranial tumors, brain malformations, as well as disturbances in cerebrospinal fluid production and drainage. However, regardless of etiology, ventriculoperitoneal (VP) shunt is a very common procedure that is used to release high intracranial pressure in those patients. As with any other invasive procedure, VP shunt placement carries numerous complications, including infections, which will either require antibiotics treatment, shunt revisions, or, in some cases, it may also result in intracranial bleeding and hematoma formation [1-2]. Ascites secondary to VP shunt is another very rare but important complication that is not well-understood.

Although the majority of ascites results from various liver pathologies, the deferential is quite varied. Cases of VP shunt-associated ascites are extremely rare and reports in the literature are few and far between. The majority of these publications report ascites development in children, however, it may also occur in adults with VP shunts as demonstrated in this case. The exact mechanism of VP shunt-related ascites is not well-understood and its causes may vary between different age groups.

## Case presentation

**Figure 1:**
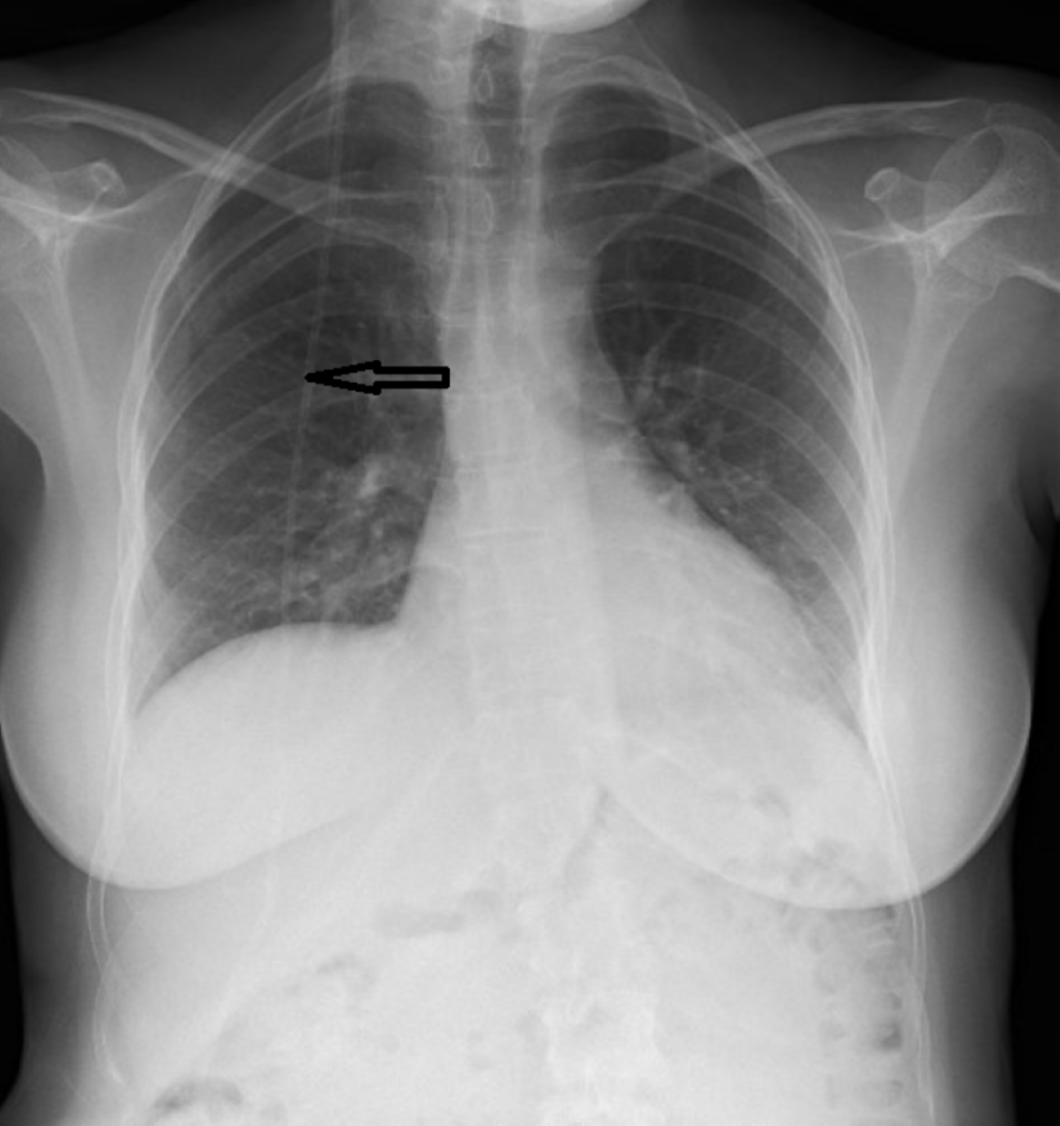
Chest X-ray Black arrow demonstrates ventriculoperitoneal shunt

**Figure 2:**
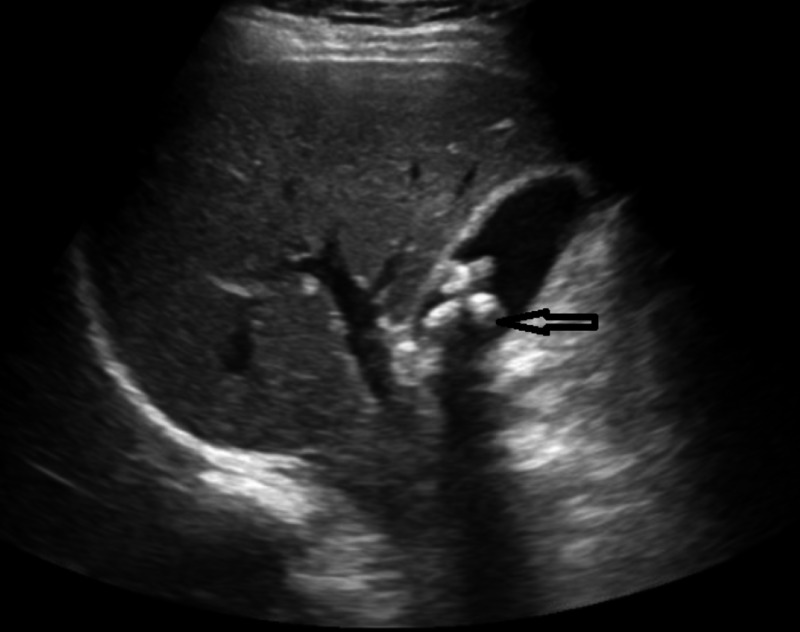
Ultrasound of liver Black arrow demonstrates cholelithiasis

**Figure 3:**
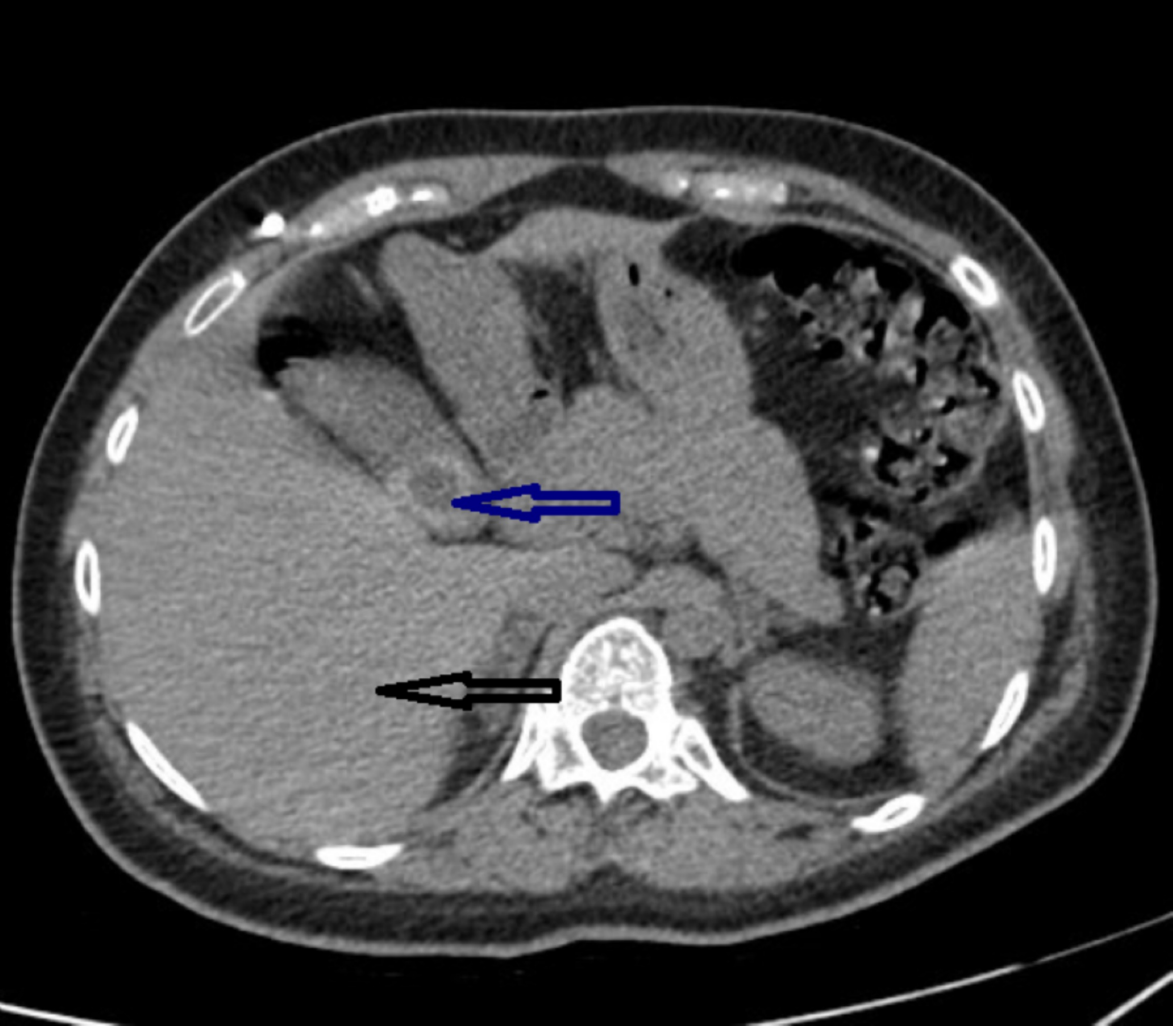
CT of abdomen Black arrow demonstrates normal, non-cirrhotic liver; Blue arrow demonstrates gallstones CT: computed tomography

A 32-year-old female with a past medical history of hydrocephalus due to brain malformation (corpus callosum agenesis), status post VP shunt placement in 1985 at three months of age presented to the hospital with abdominal pain for two days and increasing abdominal distension for the past three to four months (Figure 1). She had no significant family history and her only medication was phenobarbital 60 mg/daily for seizure prophylaxis. Vital signs were unremarkable and the only physical exam findings were abdominal distension and left lower quadrant (LLQ) tenderness to palpation. Abdominal ultrasound and abdominal computed tomography (CT) scan were performed. Findings were consistent with ascites and cholelithiasis (Figures 2-3). Subsequently, cholecystitis was ruled out with a hepatobiliary iminodiacetic acid (HIDA) scan and clinical observation. New-onset ascites workup included peritoneal fluid analysis, in addition to laboratory tests, imaging, and liver biopsy.

Paracentesis was performed at her initial presentation and had shown an ascetic albumin level of 0.6 G/DL. Trans BF2 was found in the ascetic fluid, indicating the presence of cerebrospinal fluid in the ascites. Based on this and the normal serum albumin level of 4.0 g/dL, serum-ascites albumin gradient (SAAG) > 1.1 indicated a high probability of portal hypertension and probable underlying liver disease (Tables 1-2). The results of other laboratory tests were obtained and presented in Table 2.

**Table 1:** High and low SAAG and associated pathologies SAAG: serum-ascites albumin gradient

A high gradient (SAAG >1.1 g/dL) indicates portal hypertension and suggests a nonperitoneal cause of ascites. Such conditions may include the following:	A low gradient (SAAG < 1.1 g/dL) indicates nonportal hypertension and suggests a peritoneal cause of ascites. Such conditions may include the following:
Cirrhosis	Primary peritoneal mesothelioma
Fulminant hepatic failure	Secondary peritoneal carcinomatosis
Veno-occlusive disease	Tuberculous peritonitis
Hepatic vein obstruction (ie, Budd-Chiari syndrome)	Fungal and parasitic infections (eg, Candida, Histoplasma, Cryptococcus, Schistosoma mansoni, Strongyloides, Entamoeba histolytica)
Congestive heart failure	Sarcoidosis
Nephrotic syndrome	Foreign bodies (ie, talc, cotton and wood fibers, starch, barium)
Protein-losing enteropathy	Systemic lupus erythematosus
Malnutrition	Henoch-Schönlein purpura
Myxedema	Eosinophilic gastroenteritis
Ovarian tumors	Whipple disease
Pancreatic ascites	Endometriosis
Biliary ascites	
Malignancy	
Trauma	
Portal hypertension	

**Table 2:** Laboratory results SAAG: serum-ascites albumin gradient; WBC: white blood cell, Hgb: hemoglobulin; Hct: hematocrit; MCV: mean corpuscular volume; MCH: BUN: blood urea nitrogen; AST: aspartate aminotransferase; ALT: alanine transaminase; PT: prothrombin time; INR: international normalized ratio; aPTT: activated partial thromboplastin time; AFB: acid-fast bacilli; CA-125: cancer antigen 125; IgM: immunoglobulin M; Ag: antigen; Ab: antibody; ANA: antinuclear antibodies

Lab test	Result
Albumin (ascetic fluid)	0.6 g/dL
Albumin (serum)	4.0 g/dL
Trans BF2 (ascetic fluid)	Positive
SAAG	>1.1
WBC	8.0x10³/µL
Hgb	11.9 g/dL
Hct	36.3 %
Platelets	296x10³/µL
MCV	83.3 fl
MCH	27.3 pg
BUN	7 mg/dL
Creatinine	0.47 mg/dL
Glucose	86 mg/dL
Total protein	7.3 g/dL
Alkaline phosphatase	130 IU/L
AST	13 IU/L
ALT	16 IU/L
Total bilirubin	0.2 mg/dL
PT	10.4 seconds
INR	0.99
aPTT	25.9 seconds
AFP	1.8 ng/mL
CA-125	19.2 units/mL
Hepatitis A IgM	Non-reactive
Hepatitis B core IgM	Non-reactive
Hepatitis B surface Ag	Non-reactive
Hepatitis C Ab	Non-reactive
Anti-mitochondrial antibodies	Negative
Anti-smooth muscle antibodies	Negative
ANA	Negative

The abdominal ultrasound showed a left ovarian hemorrhagic cyst, which was further investigated by MRI, which was unremarkable except for the cyst. Follow-up CT about a year later demonstrated a decrease in the size of the cyst to 1.5 cm in diameter.

The initial liver biopsy done in the hospital showed “focal and mild spotty necrosis” but was negative for fatty changes, fibrosis, or signs of portal hypertension. A repeat liver biopsy six months later showed no hepatic histopathological abnormalities.

**Figure 4:**
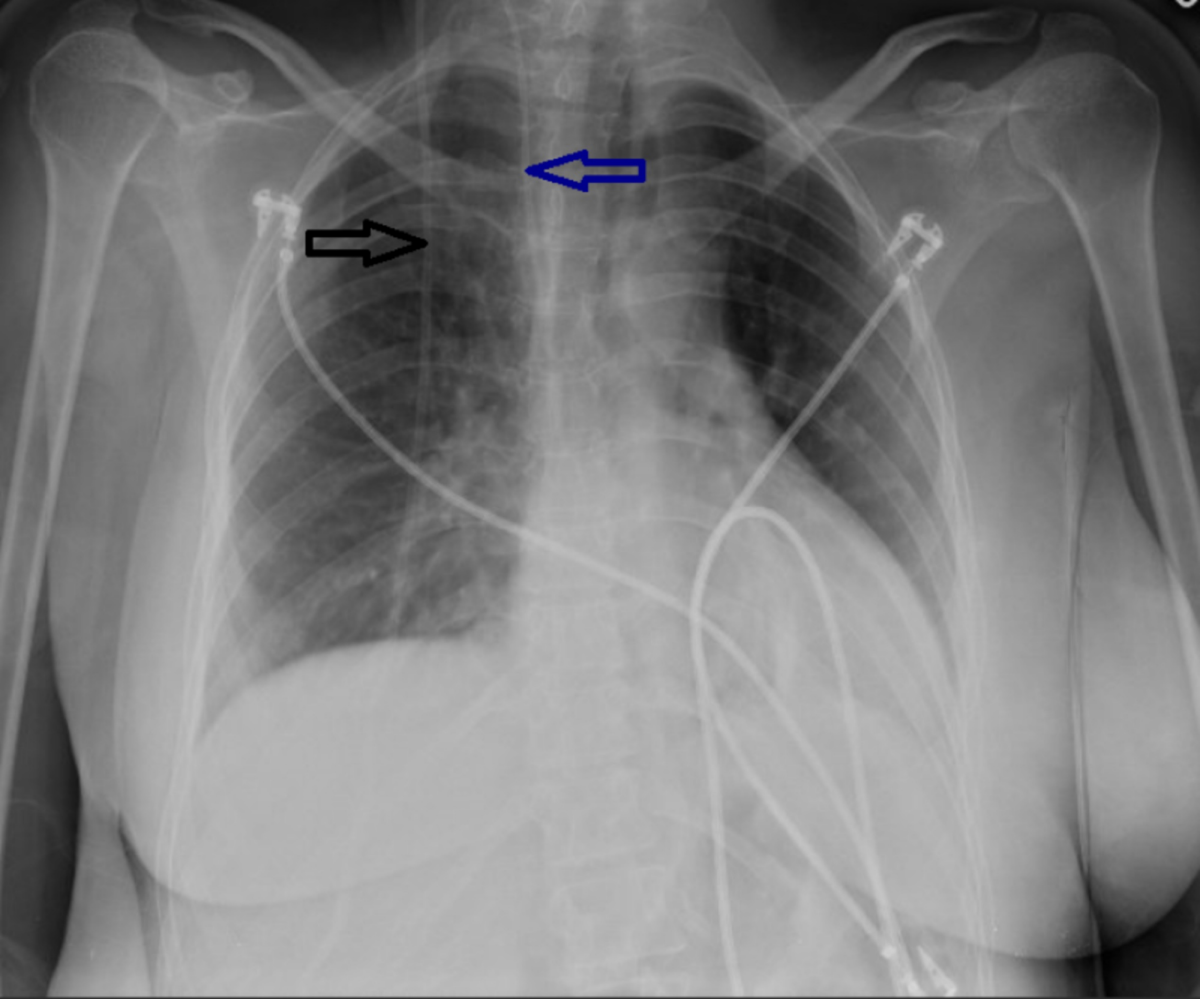
Chest x-ray. Black arrow demonstrates ventriculoperitoneal shunt Blue arrow demonstrates ventriculoatrial shunt

Initially, the patient was treated with diuretics to which there was little to no response and, ultimately, the patient required multiple paracenteses due to symptomatic ascites. Subsequently, the patient underwent ventriculoatrial (VA) shunt placement (Figure 4). Upon follow-up four months later, it was determined that the ascites had resolved.

## Discussion

A 32-year-old female with a history of hydrocephalus and VP shunt was evaluated for a new-onset ascites. An extensive workup was performed to determine the cause of the ascites. Liver pathology as the most common cause of ascites was ruled out with a liver biopsy. An incidentally found ovarian cyst was extensively evaluated for possible association with the patient’s ascites but was ultimately ruled out as a potential cause through imaging, CA-125 results, and ascetic fluid analysis. Verification of trans BF2 protein in the ascites helped identify that the cerebrospinal fluid (CSF) is the source of ascetic fluid, and in the absence of any other cause for the ascites, it was determined that the VP shunt was the cause of this patient’s ascites [3].

The majority of the cerebrospinal fluid is produced by the choroid plexuses of the lateral and fourth ventricles. CSF is produced at a rate of 600-700 ml per day and the ventricles accommodate only 25 ml. The CSF, as such, has to circulate and is absorbed across the arachnoid villi into the venous circulation and a significant amount probably also drains into lymphatic vessels around the cranial cavity and spinal canal [4]. Usually, the rate of absorption correlates with CSF pressure. Any increase in CSF production rate will increase the intracranial pressure, and a VP shunt drain will cause an increased amount of CSF flow into the peritoneal cavity and result in ascites in patients.

Hori et al., as well as Trevisi et al., proposed that hyperplasia of the choroid plexus is a potential mechanism of excessive CSF production [4-5]. However, this mechanism in the patient was ruled out by imaging.

The increased concentration of proteins in CSF from CNS tumors, infections, or any type of inflammation may increase intraperitoneal oncotic pressure and potentially lead to excessive fluid accumulation. Jamal et al. proposed this mechanism as a cause of ascites in patients in their study [6]. Since the peritoneal fluid analysis of our patient demonstrated a normal level of proteins, this mechanism of ascites formation was unlikely.

When searching for an etiology of VP shunt-related ascites, it is important to analyze it with regards to the timeline between VP shunt placement and development of the ascites. While early ascites following VP shunt placement may indicate surgery-related complications or infection, the most relevant cause several weeks to months later could be tumors and choroid plexus hyperplasia, which may play the dominant role in its development [1,7]. A careful review of the literature reveals that the majority of VP shunt-related ascites come from CNS tumors and chemotherapy, congenital hydrocephalus, choroid plexus papilloma, and infections [8]. Usually, normal skin flora tends to be the major culprit of pathogens found but rare pathogens, such as Candida albicans, Corynebacterium and Corynebacterium non-JK group, and Mycobacterium, have recently emerged as an important pathogen in the immunocompromised patients [1,6]. No infectious causes were identified that could contribute to this patient’s ascites.

The exact cause of ascites in this patient could not be established. Hemorrhage, infection, and malignancy were ruled out in this case and overproduction seemed to be the culprit. Resolving of the ascites after ventriculoatrial (VA) shunt placement suggested that VP shunt was the cause of the ascites.

## Conclusions

It is important to recognize the association of VP shunt placement and the potential development of ascites, even years later. The ascites, in this case, tends to be resistant to conventional treatment and diuretics. Treatment will usually depend on the underlying cause, such as antibiotics and potentially shunt removal for infections, or it may require switching to ventriculoatrial (VA) shunt in cases of overproduction or surgical intervention for tumors.
